# Four broad temperament dimensions: description, convergent validation correlations, and comparison with the Big Five

**DOI:** 10.3389/fpsyg.2015.01098

**Published:** 2015-08-03

**Authors:** Helen E. Fisher, Heide D. Island, Jonathan Rich, Daniel Marchalik, Lucy L. Brown

**Affiliations:** ^1^The Kinsey Institute, Indiana UniversityBloomington, IN, USA; ^2^Department of Psychology, Pacific UniversityForest Grove, OR, USA; ^3^Department of Psychology, California Southern UniversityIrvine, CA, USA; ^4^Department of Urology, Georgetown University HospitalWashington, DC, USA; ^5^Department of Neurology, Einstein College of MedicineNew York, NY, USA

**Keywords:** temperament, personality, traits, measurement, neurochemistry

## Abstract

A new temperament construct based on recent brain physiology literature has been investigated using the Fisher Temperament Inventory (FTI). Four collections of behaviors emerged, each associated with a specific neural system: the dopamine, serotonin, testosterone, and estrogen/oxytocin system. These four temperament suites have been designated: (1) Curious/Energetic, (2) Cautious/Social Norm Compliant, (3) Analytical/Tough-minded, and (4) Prosocial/Empathetic temperament dimensions. Two functional magnetic resonance imaging (fMRI) studies have suggested that the FTI can measure the influence of these neural systems. In this paper, to further the behavioral validation and characterization of the four proposed temperament dimensions, we measured correlations with five variables: (1) gender; (2) level of education; (3) religious preference; (4) political orientation; (5) the degree to which an individual regards sex as essential to a successful relationship. Subjects were 39,913 anonymous members of a US Internet dating site and 70,000+ members in six other countries. Correlations with the five variables characterize the FTI and are consistent with mechanisms using the proposed neuromodulators. We also report on an analysis between the FTI and the NEO-Five Factor Inventory, using a college sample (*n* = 215), which showed convergent validity. The results provide novel correlates not available in other questionnaires: religiosity, political orientation, and attitudes about sex in a relationship. Also, an Eigen analysis replicated the four clusters of co-varying items. The FTI, with its broad systems and non-pathologic factors complements existing personality questionnaires. It provides an index of some brain systems that contribute to temperament, and may be useful in psychotherapy, business, medicine, and the legal community.

## Introduction

It is estimated that 40–60% of the observed variance in personality is due to characteristics of temperament (Cloninger et al., 1993; Bouchard, 1994; Loehlin et al., 1998; Robins, 2005). Temperament is a heritable pattern of cognition, emotion, motivation, and behavior influenced by experience (Terracciano et al., 2005; Roberts and Mroczek, 2008) but largely stable across the lifespan (Bouchard, 1994; McCrae et al., 2000; Roberts and DelVecchio, 2000). According to Rothbart et al. (2000), “Temperament arises from our genetic endowment. It influences and is influenced by the experience of the individual, one of the outcomes is the adult personality.” Although some theorists argue that there is no hard distinction between the two constructs of personality and temperament (McCrae et al., 2000), elements of temperament traditionally include behavioral dispositions from childhood to adulthood, observable in preverbal infants and generalizable to non-human animals (Rothbart et al., 2000; Clark, 2005).

Many psychologists have investigated the physiological foundations of temperament (Eysenck and Eysenck, 1985; Cloninger, 1987, 2000; Depue et al., 1994; Gray and McNaughton, 2000; Davis et al., 2003; Zuckerman, 2005). But almost all of these models (including the NEO-PI) were initially constructed from linguistic and/or behavioral studies. As temperament is biologically based, we reasoned that constructing a temperament measure directly from data on brain architecture and physiology may elucidate core aspects of human temperament, at a broader level that might reduce crossover found among traits in other models. Dopamine has been found to be associated with both Extraversion and Openness to Experience. Previous studies and evidence presented here suggests that the Curious/Energetic scale of the Fisher Temperament Inventory (FTI) may include both and use the dopamine system (Brown et al., 2013). Also, existing measures of personality and temperament use some pathological dimensions such as: Psychoticism (Eysenck and Eysenck, 1985), Neuroticism-Anxiety (Costa and McCrae, 1992; Zuckerman, 1995), and Aggression-Hostility (Zuckerman, 1995), language that implies dysfunction. Thus the FTI has a novel physiological and behavioral focus that provides new broad dimensions.

According to Funder (2001) there is still the question of whether the “Big Five subsume all there is to say about personality. The answer is almost certainly no: whereas almost any personality construct can be mapped onto the Big Five, you cannot derive every personality construct from the Big Five.” This appears to be particularly true for aspects of temperament such as empathy, something not necessarily tied to agreeableness, as we report here. In fact, Big Five research has also identified a higher-order factor structure, or metatraits (see DeYoung and Gray, 2009) designated as stability and plasticity. Metatraits may be particularly useful a broad physiological factor structure may also be especially useful to understand personality and temperament. As researchers have noted, “…investigations must be integrated with knowledge of how personality is organized at the broadest levels, where large neural networks and broadly acting neuromodulators are likely to be important across situations” (DeYoung and Gray, 2009).

Even though there are effective and useful measures already available, we saw a need for an inventory that would be more generally descriptive of non-pathological behaviors shown in everyday, normal life, based on basic physiological influences on behavior, and easily understood and applied by individuals in a wide range of disciplines. A biological basis for The Five Factor theory has been assumed (McCrae and Costa, 1999) and physiological correlates have been found; a number of studies have correlated personality measures using biological methods like behavioral genetics (Plomin et al., 1990), epigenetics (Bussell et al., 1999) and neuroimaging (Canli et al., 2002; DeYoung et al., 2010; Haas et al., 2015). However, to our knowledge the FTI is the first measure of temperament designed directly from brain science and then tested using functional magnetic resonance imaging (fMRI), and partially validated via two fMRI studies (Brown et al., 2013), rather than the reverse of finding physiological correlates for traits established by non-biological means. The physiological hypothesis preceded the physiological tests. Further, no existing personality or temperament measure focuses on all four of these broad brain systems: the dopamine, serotonin, testosterone, and estrogen/oxytocin systems, central neural pathways characteristic of all mammalian and avian species. The ultimate purposes of the above investigations are: (1) To identify biologically based behaviors associated with *variations* in basic, non-pathologic temperament and, (2) using the FTI as an index of human variation in broad basic neural systems and behaviors, develop another useful tool to choose from, for use in psychotherapy, business, medicine, and the legal community.

Five Factor personality models have been widely used in industrial and organizational psychology and business to predict job satisfaction and performance. For example, low Neuroticism scores are predictive of less professional fulfillment (Judge et al., 2002). Despite occupational variability, Conscientiousness is consistently predictive of job performance (Barrick and Mount, 1991). But the domains of Agreeableness, Openness to Experience, and Extraversion are constrained in their predictive ability to those occupations (sales) that require greater social competency and persuasion (Barrick and Mount, 1991) and therefore may be less predictive of job performance across occupations. Among the Five Factor personality measures (NEO Personality Inventory; Costa and McCrae, 1992; Big Five Inventory; Goldberg, 1993; International Personality Item Pool-Five Factor Model; Goldberg et al., 2006; Ten Item Personality Inventory; Gosling et al., 2000) Neuroticism has not been shown to predict competency or business success. Other models of personality, like the six-factor HEXACO (i.e., Honesty–Humility, Emotionality, Extraversion, Agreeableness, Conscientiousness, and Openness to Experience) framework (Ashton and Lee, 2007) may have more value in organizational settings, due to its inclusion of a sixth facet, Honesty–Humility, a factor demonstrated to predict integrity and ethical decision-making beyond other measures of the traditional Big Five (Lee et al., 2008). However, additional factors may be useful in business to predict team compatibility, client/consultant compatibility, tough-mindedness and innovative thinking, as well as compatibility in a range of personal relationships.

To construct this new temperament inventory, we first extracted from a literature review traits linked with any neurochemical system. Four suites of characteristics emerged; each suite was associated primarily with one of four broad brain systems: the (1) dopamine; (2) serotonin; (3) testosterone; and (4) estrogen/oxytocin systems (Fisher et al., 2010a,b; Brown et al., 2013). Using factor analysis, we developed a 56-item questionnaire, the FTI, and determined that these four clusters, based on the physiological literature, could be identified (Fisher et al., 2010b). We proposed four temperament dimensions and referred to them respectively as the Curious/Energetic scale; the Cautious/Social Norm Compliant scale; the Analytical/Tough-minded scale; and the Prosocial/Empathetic scale on the FTI (Fisher et al., 2010b; Brown et al., 2013). Then, in two experiments using fMRI, scores on each of the four FTI scales were significantly correlated with activations in some of the predicted brain regions, including known dopamine-rich regions and regions influenced by sex hormones (Brown et al., 2013).

In the present study we further characterize the FTI with three new investigations: (1) we examine its correlations with five demographic variables. The five variables were chosen because they are known to have associations with biological mechanisms; included are: gender; religiosity; level of education; political orientation; and attitude regarding the importance of sex in a relationship. (2) We carry out a convergent validity analysis with an established measure of personality, the short form of the NEO-Personality Inventory Revised, the NEO-Five Factor Inventory (NEO-FFI; Costa and McCrae, 1992). (3) We replicate our factor analysis results of the FTI with another method, Eigen Analysis.

The purpose of the present investigation is: (1) To determine any possible correlations between these four broad temperament dimensions and five demographic variables know to have biological components; (2) To expose additional facets of the FTI by comparing it with a well known psychometric measure, thus further defining these proposed four broad temperament dimensions.

### Predictions

Based on sex differences associated with bound and bioavailable testosterone, estrogen, and oxytocin, we predicted that men would score higher on the Analytic/Tough-minded scale, while women would score higher on the Prosocial/Empathetic scale. For example, endogenous testosterone is associated with diminished emotion recognition, eye contact and social sensitivity (Lutchmaya et al., 2002); and reduced empathy (Knickmeyer et al., 2006), while prenatal estrogen priming is associated with agreeableness, cooperation, theory of mind (Baron-Cohen, 2003), and empathy and nurturing (Knickmeyer et al., 2006). More references for the predictions and rationale for all the predictions can be found in Section “Materials and Methods.”

We anticipated that Level of Education would be correlated with the Curious/Energetic scale because attaining a higher academic degree requires elevated curiosity, motivation and energy (Subotnik et al., 2011), traits linked in the biological literature with the dopamine system (Depue and Collins, 1999; Zuckerman and Kuhlman, 2000; Wacker et al., 2006).

We predicted that individuals scoring highest on the Cautious/Social Norm Compliant scale would be significantly more likely to be members of an organized, conventional religious group, as this is consistent with genetic data associating aspects of the serotonin system with religiosity (Lorenzi et al., 2005; Ott et al., 2005) and traditionalism (Golimbet et al., 2004).

We anticipated that participants who scored highest on the Cautious/Social Norm Compliant scale would be more politically conservative because self-reported conservatives in other western countries score higher than self-reported liberals on scales of respect for authority and tradition (Graham et al., 2009), characteristics of the proposed Cautious/Social Norm Compliant dimension. Also, traditionalism is linked in the biological literature with aspects of the serotonin system (Golimbet et al., 2004). We also hypothesized that participants who scored highest on the Prosocial/Empathetic scale would be significantly more liberal in their political views, because self-reported liberals in dozens of countries score higher than conservatives on scales of caring/nurturance (Graham et al., 2009), traits associated in the biological literature with the estrogen and oxytocin systems (Knickmeyer et al., 2006).

Last, elevated activity in the testosterone and dopamine systems is widely associated with elevated sex drive (Bagatell et al., 1994; Meston and Frohlic, 2000), so we anticipated that those individuals with a higher sex drive would be more likely to regard sex as important to a successful partnership. Thus, we predicted that scores on both the Analytical/Tough-minded scale and the Curious/Energetic scale would positively correlate with the statement, “Sex is an essential part of a successful relationship.” Further, since higher central serotonin regularly suppresses sexual desire and sexual function (Rosen et al., 1999), we also predicted that higher scores on the Cautious/Social Norm Compliant scale would negatively correlate with the statement, “Sex is an essential part of a successful relationship,” because individuals with a lower sex drive might regard sex as less important to a successful partnership.

We undertook the comparison between the FTI and the NEO-FFI for two reasons: (1) the NEO PI-R and NEO FFI are widely used as psychometric comparators for temperament and personality instrument development and validation; so this comparison might further the understanding of the characteristics likely to be associated with each of the four proposed temperament dimensions of the FTI; and (2) all of the scales of the NEO PI-R and NEO FFI have shown modest heritability (Plomin and Caspi, 1999) and the FTI is designed to measure heritable behavior patterns associated with temperament. Positive correlations would be evidence that it could measure heritable behavior patterns, also. Divergent findings might point out the unique contributions of the FTI.

Regarding our comparison between the FTI and the NEO-FFI, we had three predictions: (1) that scores on the Curious/Energetic scale of the FTI would correlate with those on the Open to New Experiences scale of the NEO-FFI because both scales have been associated with exploratory behavior, novelty-seeking and curiosity (Costa and McCrae, 1992; Depue and Collins, 1999); (2) that scores on the Cautious/Social Norm Compliant scale of the FTI would correlate with the Conscientious scale of the NEO-FFI because both the NEO-FFI domain of Conscientiousness and the Cautious/Social Norm Compliant scale on the FTI attempt to measure self-control and self-regulation (Costa and McCrae, 1992), as well as the desire to plan and organize (DeYoung and Gray, 2009); (3) that higher scores on the Analytical/Tough-minded scale of the FTI would correlate negatively with high scores on the Agreeable scale of the NEO-FFI because tough-mindedness is likely to be the opposite of tender-mindedness, a trait in the Agreeableness domain of the NEO-FFI.

We had no predictions regarding a correlation between the Neuroticism scale of the NEO-FFI and any scale of the FTI because the FTI does not attempt to measure neuroticism; nor did we have any hypotheses regarding a correlation between the Extraversion scale of the NEO-FFI and any scale of the FTI because the FTI does not attempt to measure extraversion.

## Materials and Methods

### Online Participants

To test a relatively large, international, non-college population and thus offer statistical power and generalizability, this study used archived data from the commercial websites Chemistry.com and Match.com. Consequently, our samples consisted of anonymous survey data. Participant informed consent was obtained through the U.S. dating websites Chemistry.com^®^ and Match.com^®^ during the registration process when members acknowledged and accepted the privacy statement and third party data-release policies. Given informed consent was obtained by the primary party, not the researchers, Rutgers University and Pacific University Institutional Review Board did not require that we obtain or solicit for *post hoc* informed consent to use the online survey data.

#### North American Sample

A sample of 17,392 men and 22,521 women (*N* = 39,913) were solicited for their participation in this study through the U.S. Internet dating site, Chemistry.com^®^. There were no inclusion or exclusion criteria, the sample consisted of members or visitors to the dating website and required that all individuals were of 18 years of age, and were not currently in a relationship and were looking for someone to date.

The data were collected from test-takers over three consecutive weeks at Chemistry.com^®^. Participants ranged in age from 18 to 88 years (*M* = 37.0; *SD* = 12.6); 89.6% sought an opposite sex partner. The geographic range included all 50 of the United States and all 13 provinces in Canada, including urban, suburban, and rural populations. Over half of the participants did not report their ethnic identity (*n* = 23,530; 59%); those who did (*n* = 16,383; 41%) were calculated as part of the whole population. Participants who reported ethnicity included: 1,310 (8.0%) African-Americans; 12,505 (76.3%) self-reported Euro-Americans [i.e., Caucasians; 359 (2.2%) were self-reported as the broad descriptor, Asian; 861 (5.3%) participants were Latino or Latina; 59 (0.36%) participants reported a “Middle Eastern” ethnic identity; 103 (0.63%) were Native American; 262 (1.6%) simply selected the innocuous category of “Other” and finally 881 (5.4%) participants reported mixed ethnic identities]. In addition to the ethnic identity demographic information, 4,154 (10.4%) participants reported seeking same-sex partners while the remaining 35,759 (89.6%) sought opposite sex-partners.

#### International Sample

Individuals took translated versions of the FTI questionnaire on related Internet dating sites in six other countries. Included in the international sample were participants from Match.com^®^ sites in: Germany (*n* = 12,498); France (*n* = 12,713); Spain (*n* = 12,652); Sweden (*n* = 12,722); Australia (*n* = 12,498), and Japan (*n* = 11,770). Translated questionnaires were used in all countries except Australia, where the U.S. measure was administered.

#### Eigen Analysis Sample

For the Eigen analysis, a North American sample of 100,000 different anonymous members of and visitors to the same Internet dating site was used. This different sample was used because the Eigen Analysis was carried out at a different time from the other studies. There were no inclusion or exclusion criteria, except that all individuals were single and not in a partnership. Participants came from all 50 American states and 13 Canadian provinces and territories. Participants ranged in age from 18 to 88 years (*M* = 39.6, *SD* = 13.4); 52% were female; 92.8% sought an opposite sex partner. The geographic range included urban, suburban, and rural populations. Site employees regularly check the composition of members and it did not differ over the time period during which the studies discussed in this paper were carried out.

### College Student Participants

The criterion validity study of the FTI and the NEO-FFI used self-report data from 81 men (*M_age_* = 21.77 years; SD*_age_* = 5.41) and 109 women (*M_age_* = 20.18 years; SD*_age_* = 4.61) enrolled in undergraduate and professional programs at Pacific University (*N* = 215). For those students who had tied temperament dimensions (*n* = 24) or who did not complete the survey (*n* = 1), their data was omitted for a final sample of 190 students. All participants signed an informed consent disclosure, and were provided $25 remuneration for their involvement.

### Materials

The 56-items FTI consists of the four broad temperament dimensions: Curious/Energetic; Cautious/Social Norm Compliant; Prosocial/Empathic; and Analytical/Tough-Minded; each category has 14-items. The response options reflect a four option, Likert-like agreement scale with a score of 0 for “strongly disagree,” 1 for “disagree” 2 for “agree” and 3 for “strongly agree” (Fisher et al., 2010b).

The questions were designed using the biological literature. For example, activity in the dopamine system has been correlated with novelty and thrill and adventure seeking, boredom susceptibility and disinhibition (Cloninger et al., 1991; Comings et al., 2000; Zuckerman and Kuhlman, 2000), stamina, motivation and achievement striving (Depue and Collins, 1999; Wacker et al., 2006); abstract intellectual exploration (DeYoung et al., 2002); cognitive flexibility (Ashby et al., 1999); curiosity (Zuckerman and Kuhlman, 2000); verbal and non-linguistic creativity, idea generation (Flaherty, 2005; Reuter et al., 2006), low anxiety (Laakso et al., 2003) and poor introspection (Cloninger et al., 1991). The Curious/Energetic scale included statements such as, “I am always doing new things,” “My friends would say I am very curious,” and “I have more energy than most people.”

Activity in the serotonin system has been correlated with adherence to social norms (i.e., conventionalism; Golimbet et al., 2004); self control and self-regulation (Linnoila et al., 1994; Manuck et al., 1998); sociability (Golimbet et al., 2004); harm avoidance (Parks et al., 1998; Golimbet et al., 2004); precision and interest in details (Cloninger et al., 1991); conscientiousness (Manuck et al., 1998; DeYoung et al., 2002, 2010; DeYoung and Gray, 2009); cooperation (Bilderbeck et al., 2014) managerial skills (e.g., cooperation, reduced commands and reduced autonomous problem-solving; Knutson et al., 1998); figural and numeric creativity (Reuter et al., 2006); and self-transcendence (e.g., religiosity; Lorenzi et al., 2005; Ott et al., 2005). The Cautious/Social Norm Compliant scale included statements such as: “People should behave in ways that are morally correct,” “My friends and family would say I have traditional values,” and “In general, I think it is important to follow rules.”

Prenatal testosterone priming is linked with enhanced visual-spatial perception, mathematical skills, musical aptitude, aggressiveness, and compromised verbal fluency (Geschwind and Galaburda, 1985; Manning et al., 2001; Manning, 2002). Endogenous testosterone is also associated with enhanced attention to detail, focused attention (Knickmeyer et al., 2005); diminished emotion recognition, eye contact and social sensitivity (Lutchmaya et al., 2002); and reduced empathy (Knickmeyer et al., 2006). Characteristics correlated with activational testosterone (i.e., post-natal exposure) include enhanced self-assurance (Zilioli and Watson, 2013), candid and assertive communication (Nyborg, 1994; Archer, 2006; Guinn Sellers et al., 2007), sensitivity to social dominance and drive for rank (Mazur et al., 1997; Eisenegger et al., 2011), and emotional comportment (Dabbs, 1997). Questions in the Analytical/Tough-minded scale include, “I enjoy competitive conversations,” “I am more analytical and logical than most people,” and “I understand complex machines easily.”

In contrast, prenatal estrogen priming is associated with contextual thinking (Baron-Cohen et al., 2005), linguistic skills (Rosenberg and Park, 2002), agreeableness, cooperation, theory of mind (Baron-Cohen, 2003), and empathy and nurturing (Knickmeyer et al., 2006). In addition, activational estrogen (post-natal exposure to estrogen) is positively correlated with generosity and trust (Kosfeld et al., 2005), agreeableness (Treleaven et al., 2013) the drive to make social attachments (Carter, 1998; Edelstein et al., 2010), and heightened memory for emotional experiences (Canli et al., 2002). Similarly, oxytocin is associated with prosocial behavior (Carter, 1998) including trust (Zak et al., 2007), prosody (Barraza and Zak, 2009), introspection and perspective-taking (Domes et al., 2007). The Prosocial/Empathetic scale included statements such as: “I like to get to know my friends deepest needs and feelings,” “I highly value deep emotional intimacy in my relationships,” and “Regardless of what is logical, I generally listen to my heart when making important decisions.”

The Cronbach’s alpha coefficients for the U.S. sample were: 0.79 for both the Curious/Energetic and Cautious/Social Norm Compliant constellations; 0.80 for the Analytical/Tough-minded subscale; and 0.78 for the Prosocial/Empathetic scale.

#### The NEO-Five Factor Inventory

The NEO-FFI is a 60-item Five-Factor personality inventory (12 questions/domain) based on the longer 240-item measure. Because the FTI is a 56-item questionnaire, the shorter NEO-FFI was regarded as a more suitable comparator than the longer NEO PI-R. Like the FTI, the NEO-FFI is scored using a Likert-like scale with the following internal consistency coefficients: 0.79 for the domains of Neuroticism and Extraversion, 0.80 for Openness to Experience, 0.75 for Agreeableness, and 0.83 for Conscientiousness (Costa and McCrae, 1992).

### Statistical Analysis

#### False Discovery Rate

The Bonferroni correction is commonly applied to multiple inferential statistical tests and controls the familywise error rate. Benjamini and Hochberg (1995) argue that this procedure is too conservative, and risks Type II error, failure to detect real effects. They propose an alternative procedure, the False Discovery Rate (FDR), which is more powerful, and which controls for the expected proportion of falsely rejected hypotheses. Thus we used FDR for the 144 comparisons across all the comparisons we made in this study, including the comparison with the NEO-FFI, and 0.05 as the critical *p*-value.

#### Correlation Measures

Pearson *r* correlations (two-tailed) between FTI scores and responses to three variables were carried out. The three variables were: (1) education, (2) political orientation, and (3) the extent to which one regards sex as an essential part of a successful relationship. Education level was coded as (1): Not a high school graduate; (2): High school graduate; (3): Some college; (4): Associate’s degree; (5): Bachelor’s degree; (6): Graduate school; (7): Doctorate. Participants were asked to describe their political orientation and given the options: “Very liberal,” Liberal,” “Conservative,” “Ultra conservative,” “Other.” To measure the degree to which one regards sex as an essential part of a successful relationship, participants rated their level of agreement to the statement, “Sex is an essential part of a successful relationship” by selecting one of four options: “Not at all,” “A little,” “Quite a bit,” “Very much so.”

#### *T*-Tests

*T*-tests were carried out to compare men and women on each dimension, and to compare “religious” and “non-religious.” Participants were classified as “religious” if they specified that they identified with a particular religion. Participants were classified as not religious if they chose the categories “atheist,” “agnostic,” “spiritual but not religious,” or “not religious.”

When *t*-tests were completed, tests for homogeneity of variance were performed, and tests for unequal variance were used where applicable. The test scores for each of the four scales showed a normal distribution, with a small deviation from normality at the low end of the scores. This was not a concern because *t*-tests are considered to be robust with respect to the normality assumption, particularly with large samples (Sawilowsky and Blair, 1992).

#### Effect Sizes

The odds ratio (OR 0.5 [95% Confidence Interval]), was calculated to estimate effect size in a large population. Pearson *r* correlations are also an effect size. Other effect sizes (η^2^) were calculated for raw mean score comparisons. Effect size calculations are important in a study with a large number of participants, to help assess the functional significance of the statistical significance.

Questionnaire scores in the text are reported as mean ± SD and SE of the mean. Both measures of variability alert the reader to the variability in the data for this large sample, and the statistical significance of the relatively small effects. The figures show mean ± SE.

#### Eigen Analysis

To replicate our basic questionnaire clustering results with a method different from factor analysis, an Eigen analysis on standardized scores was used. Software scripts in the R programming language were used on the open access Galaxy platform (Goecks et al., 2010). A topologic algorithm was used that treats each survey item as an independent attribute (vector) and employs Eigen analysis to identify distinct topologies. Each point in space (see **Figure 4**) demonstrates varied combinations of temperament affinities and disaffinities. Linear regression was used to compare the relative positions of each item in each dimension. To determine the stability and reproducibility of the identified population temperament structure using this method, the same analysis was performed on two independent, randomly sorted subsets of 50,000 responses.

## Results

### Sex Composition

Among the *North American Sample*, 26.0% of the men scored highest for Analytic/Tough-minded; while only 9.7% of women scored highest on this proposed temperament dimension (**Table 1**; OR = 3.3 [3.1–3.5]; *χ^2^* = 1617, *p* = 1 × 10^-200^). In addition, 35.1% of the women scored highest for the Prosocial/Empathetic scale, while significantly fewer men scored highest on this proposed temperament dimension: 20.3% (OR = 2.1 [2.0–2.2] *χ^2^* = 918, *p* = 1 × 10^-200^). For the Curious/Energetic and Cautious/Social Norm Compliant scales, the odds ratios for the difference between men and women were close to 1.0 (**Table 1**), showing very small differences.

**Table 1:** Percent men and women with highest score for FTI characteristics.

Sample type	% Men	% Women	Odds^∗^	Lower	Upper	χ^2^	*p*
**Pacific University (*n* = 190)**							
Analytic	24.7	5.5	5.66	0.07	0.61	8.60	0.003^∗^
Prosocial	17.3	35.8	2.67	1.34	8.37	6.89	0.008^∗^
Curious	18.5	16.5	1.15	0.47	2.93	0.12	0.731
Cautious	39.5	42.2	1.12	0.26	1.76	0.65	0.42
**North America (*n* = 34,831, 44% men)**							
Analytic	26.0	9.7	3.26	3.07	3.46	1617.69	<1.0E-200^∗^
Prosocial	20.3	35.1	2.13	2.02	2.23	918.47	<1.0E-200^∗^
Curious	25.3	23.8	1.08	1.03	1.14	10.38	0.0013^∗^
Cautious	28.5	31.4	1.15	1.10	1.20	34.58	4.08E-09^∗^
**Australia (*n* = 12,498, 53% men)**							
Analytic	26.2	10.2	3.21	2.90	3.55	555.71	7.19E-123^∗^
Prosocial	20.8	41.0	2.65	2.45	2.86	601.09	9.67E-133^∗^
Curious	17.6	18.1	1.03	0.94	1.13	0.38	0.5379
Cautious	31.2	34.5	1.16	1.08	1.25	15.28	9.27E-05^∗^
**France (*n* = 12,713, 51% men)**							
Analytic	40.6	19.9	2.75	2.54	2.98	639.92	3.47E-141^∗^
Prosocial	6.2	16.8	3.07	2.73	3.47	358.47	6.07E-80^∗^
Curious	24.6	20.5	1.26	1.16	1.37	30.06	4.20E-08^∗^
Cautious	38.6	32.7	1.30	1.21	1.39	49.15	2.37E-12^∗^
**Germany (*n* = 12,388, 52% men)**							
Analytic	27.7	8.6	4.07	3.66	4.52	755.06	3.19E-166^∗^
Prosocial	23.9	50.0	3.19	2.96	3.45	911.66	2.86E-200^∗^
Curious	23.1	25.6	1.15	1.06	1.24	10.65	0.0011^∗^
Cautious	18.3	22.8	1.32	1.21	1.44	38.65	5.07E-10^∗^
**Japan *(n* = 11,770, 72% men)**							
Analytic	32.8	10.8	4.05	3.60	4.56	594.56	2.56E-131^∗^
Prosocial	25.8	52.2	3.15	2.90	3.42	752.49	1.15E-165^∗^
Curious	18.6	16.6	1.15	1.03	1.27	6.61	0.0101^∗^
Cautious	18.5	24.8	1.46	1.32	1.61	54.87	1.29E-13^∗^
**Spain (*n* = 12,652, 59% men)**							
Analytic	47.2	24.8	2.71	2.51	2.93	652.20	7.44E-144^∗^
Prosocial	19.6	42.2	3.00	2.77	3.25	764.55	2.76E-168^∗^
Curious	14.3	12.7	1.15	1.04	1.27	6.99	0.0082^∗^
Cautious	18.7	20.6	1.13	1.03	1.23	6.77	0.0093^∗^
**Sweden (*n* = 12,722, 56% men)**							
Analytic	39.9	15.1	3.74	3.43	4.08	943.68	<1.0E-200^∗^
Prosocial	15.8	38.3	3.30	3.04	3.59	828.58	3.30E-182^∗^
Curious	21.0	19.8	1.08	0.99	1.18	2.93	0.0871
Cautious	25.7	24.5	1.06	0.98	1.15	2.23	0.1354

*^∗^Significant using FDR and p-value 0.05 criterion.*

In the *International Sample*, the results were similar. Odds ratios ranged from 2.6 to 4.1 for the difference between men and women in the Analytic/Tough-Minded and Prosocial/Empathetic scales; odds ratios ranged from 1.0 to 1.5 for the difference between men and women on the other scales (**Table 1**).

In the *Pacific University Sample*, the results were again similar: 24.7% of men scored higher than women on the Analytic/Tough-Minded scale (OR = 5.5 [0.07–6.1] **Table 1**) while on the Prosocial/Empathetic scale 36.7% of the women scored higher than men (OR = 2.5 [1.3–8.3]). The other two scale comparisons (Curious/Energetic and Cautious/Social Norm Compliant) showed odds ratios close to 1.0 (**Table 1**) and were not statistically different.

**FIGURE 1:**
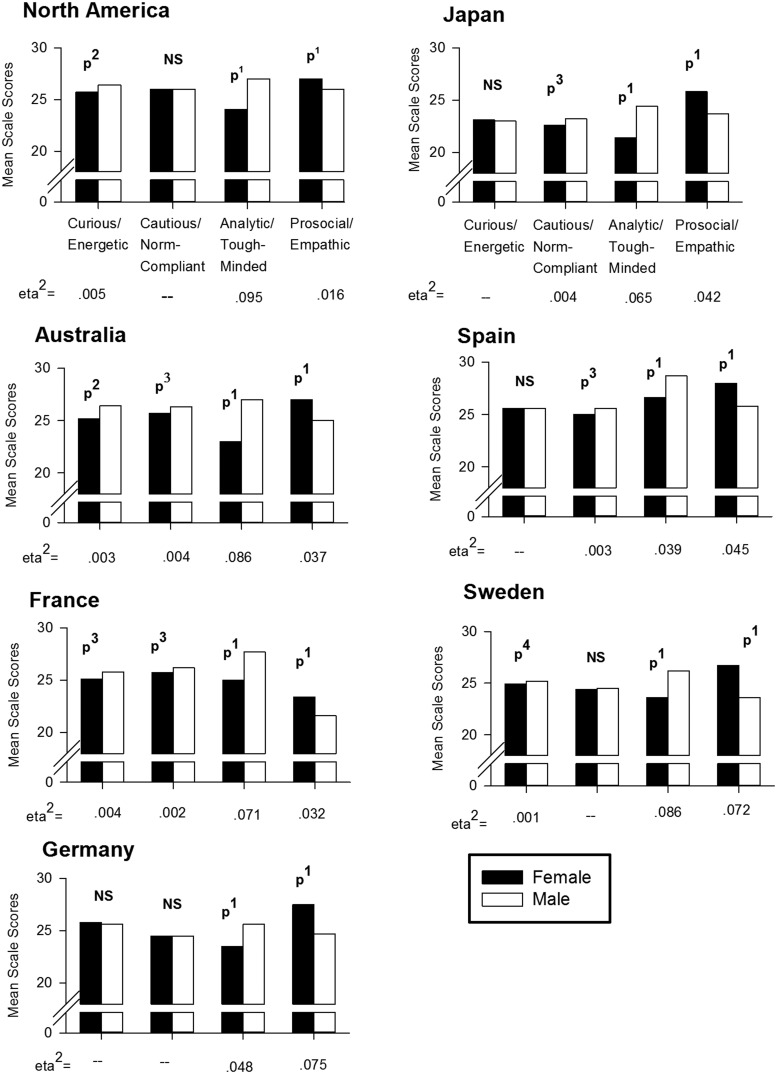
**Mean scale scores by men and women in North America and other countries**. Sex consistently played a role in the scores for the Analytical/Tough-minded and Prosocial/Empathetic scales across seven countries, and less so for the other two scales. Effect size and statistical significance were greater for the Analytical/Tough-minded and Prosocial/Empathetic scales than for the other scales. *N* = 384,831 for the United States sample. *N* = ∼12,500 for each of the other countries. *p*^1^ = *p* < 1.0 × 10^-100^; *p*^2^ = *p* ≤ 1.24 × 10^-40^; *p*^3^ = *p* ≤ 6.0 × 10^-9^; *p*^4^: *p* = 0.0091. NS, not statistically significant. All effects are statistically significant using the FDR multiple comparison test at a 0.05 criterion. For SE (generally too small to see in the figure) and SD, see Results.

The raw scores for the *North American Sample* show that the men’s mean scores were higher than those for women on the Analytical/Tough-minded scale (Men: 26.8 ± 5.0, *SE* = 0.038; Women: 23.6 ± 4.9, *SE* = 0.033; η^2^ = 0.093; *t* = 63.89, *p* < 1 × 10^-150^; unequal variance: *F* = 5.00, *p* = 0.025; **Figure 1**). Women scored higher than men on the Prosocial/Empathetic scale (26.9 ± 5.0, SE = 0.033, vs. 25.6 ± 4.9, SE = 0.038; η^2^ = 0.017; *t* = 26.16, *p* = 1.37 × 10^-149^; **Figure 1**). In addition, North American men scored higher than women on the Curious/Energetic scale, but the effect size was very small (26.3 ± 4.8, SE = 0.037; vs. 25.7 ± 4.8, SE = 0.032; η^2^ = 0.004; *t* = 13.36, *p* = 1.24 × 10^-40^; **Figure 1**). The Cautious/Social Norm Compliant scale showed no sex difference (**Figure 1**).

In the other six countries investigated, the same raw mean score differences between sexes were found for the Analytical/Tough-minded and Prosocial/Empathetic scales, with small to medium effect sizes (**Figure 1**; η^2^: 0.048–0.095 for Analytic/Tough-minded; η^2^: 0.016–0.075 for Prosocial/empathetic). For the Curious/Energetic scale response, sex differences were very small or non-existent (<0.000–0.004; **Figure 1**). On the Cautious/Social Norm Compliant scale, sex differences also were small or not significant (0.0001–0.004; **Figure 1**). Thus, for the Analytic/Tough-minded and Prosocial/Empathetic scales the effect sizes were statistically significant and small to medium in all countries tested, while the other scales were not consistently different between the sexes, and any statistical effect sizes were extremely small.

### Correlation Analyses

#### Level of Education

**FIGURE 2:**
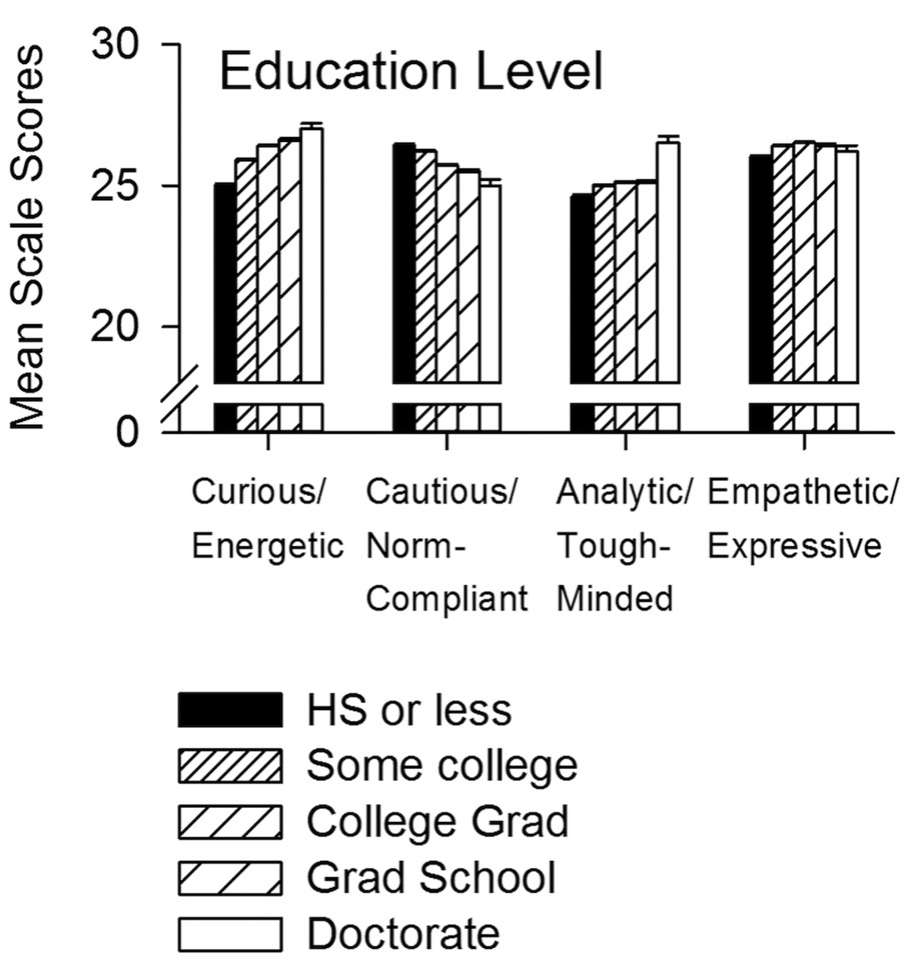
**Mean scale scores by educational level.** The Curious/Energetic scale showed the highest positive correlation with level of education (*r* = 0.099; *p* = 2.2 × 10^-87^) compared to the other scales. The other scales showed either: a negative correlation with level of education (*r* = -0.065, *p* = 3.9 × 10^-38^, Cautious/Norm Compliant); a lesser positive correlation (*r* = 0.037, *p* = 2.3 × 10^-13^, Analytical/Tough-Minded); or no correlation (*r* = 0.015, NS, Prosocial/Empathetic). All effects are statistically significant using the FDR multiple comparison test at a 0.05 criterion. For SE (generally too small to see in the figure) and SD, see Results.

Curious/Energetic scores showed the highest correlation with level of education compared to the other three scales (*r* = 0.099, *p* = 2.2 × 10^-87^, **Figure 2**). Prosocial/Empathetic scores were not significantly correlated (*r* = 0.015, NS, **Figure 2**), while other Pearson *r* correlations between scores and educational level were very small or negative (**Figure 2**): Cautious/Social Norm Compliant *r* = -0.065, (*p* = 3.9 × 10^-38^); Analytical/Tough-minded *r* = 0.037 (*p* = 2.3 x 10^-13^).

#### Religious Preference

A specific organized religion was chosen by 67.2% of participants; they were classified as religious. The other 32.8% were classified as non-religious. Among those who were religious, 35.4% were classified as Cautious/Social Norm Compliant; among those who were non-religious, 19.5% were classified as Cautious/Social Norm Compliant (OR = 2.3 [2.2–2.4], **Table 2**). The differences were smaller for the other temperament dimensions and the odds ratios ranged from only 1.1 to 1.5 (see **Table 2**).

**Table 2:** Religious and non-religious by FTI subscale.

Type (*n* = 34,831)	Percent non-religious	Percent religious	Odds ratio 95% CI	Lower	Upper	χ^2^ (df = 1)	*p*-value
Analytic/Tough-Minded	20.2%	15.1%	1.4	1.3	1.5	144.436	2.85E-33
Prosocial/Empathic	34.4%	25.8%	1.5	1.4	1.5	273.149	2.34E-61
Curious/Energetic	26.0%	23.7%	1.1	1.1	1.2	22.048	2.66E-06
Cautious/Norm Compliant	19.5%	35.4%	2.2	2.1	2.3	928.130	1.0E-200

*“Religious” indicates that the participant identified with a particular religion. “Non-religious” includes agnostic, atheist, “none,” and “spiritual but not religious.” All tests are statistically significant using FDR and p-value 0.05 criterion.*

Raw scores showed that those classified as religious scored higher than non-religious on the Cautious/Social Norm Compliant subscale (26.7 ± 4.4, SE = 0.027 vs. 24.5 ± 4.6, SE = 0.040, *p* < 1.0 × 10^-50^). The effect size was 0.048. The religious responders also scored significantly lower on the other three scales, but with very small effect sizes (*η*^2^ = 0.002–0.004).

#### Political Orientation

The mean scores and *r*-values are shown in **Table 3**. Scores positive for political conservatism were as follows: Cautious/Social Norm Compliant: *r* = 0.23 (*p* < 1 × 10^-50^); Analytical/Tough-Minded: *r* = 0.02 (*p* = 0.001). Small negative correlations were found for Curious/Energetic: *r* = -0.07 (*p* < 3.5 × 10^-7^) and Prosocial/Empathetic: *r* = -0.15 (*p* < 1 × 10^-50^, **Table 3**).

**Table 3:** Political party affiliation by subscale score means.

		Curious		Cautious		Analytic		Prosocial	
Party affiliation	N	M (SD)	SE	M (SD)	SE	M (SD)	SE	M (SD)	SE
Ultra liberal	2237	27.2 (5.0)	0.1	23.7 (5.5)	0.1	25.5 (5.7)	0.1	28.5 (5.3)	0.1
Liberal	9777	26.2 (4.8)	0.1	25.1 (4.6)	0.1	24.7 (5.2)	0.1	27.0 (5.0)	0.1
Other	18930	26.0 (4.9)	0.1	25.9 (4.4)	<0.1	25.1 (5.2)	<0.1	26.2 (5.0)	<0.1
Conservative	8545	25.5 (4.8)	<0.1	27.7 (4.2)	<0.1	25.1 (5.3)	<0.1	25.4 (4.8)	<0.1
Ultra Conservative	424	25.4 (5.7)	0.2	28.7 (5.6)	0.1	25.9 (6.0)	0.3	25.5 (5.9)	0.2
Pearson r for conservatism		-0.07		0.23		0.02		-0.15	
*p*-value for correlation		3.5 ^∗^ 10^-47^		<10^-50^		0.00107		<10^-50^	

*Full scale names: Curious/Energetic; Cautious/Norm Compliant; Analytic/Tough-Minded; Prosocial/Empathetic. All effects are statistically significant using the FDR multiple comparison test at a 0.05 criterion.*

#### Importance of “Sex as essential to a Successful Relationship”

**FIGURE 3:**
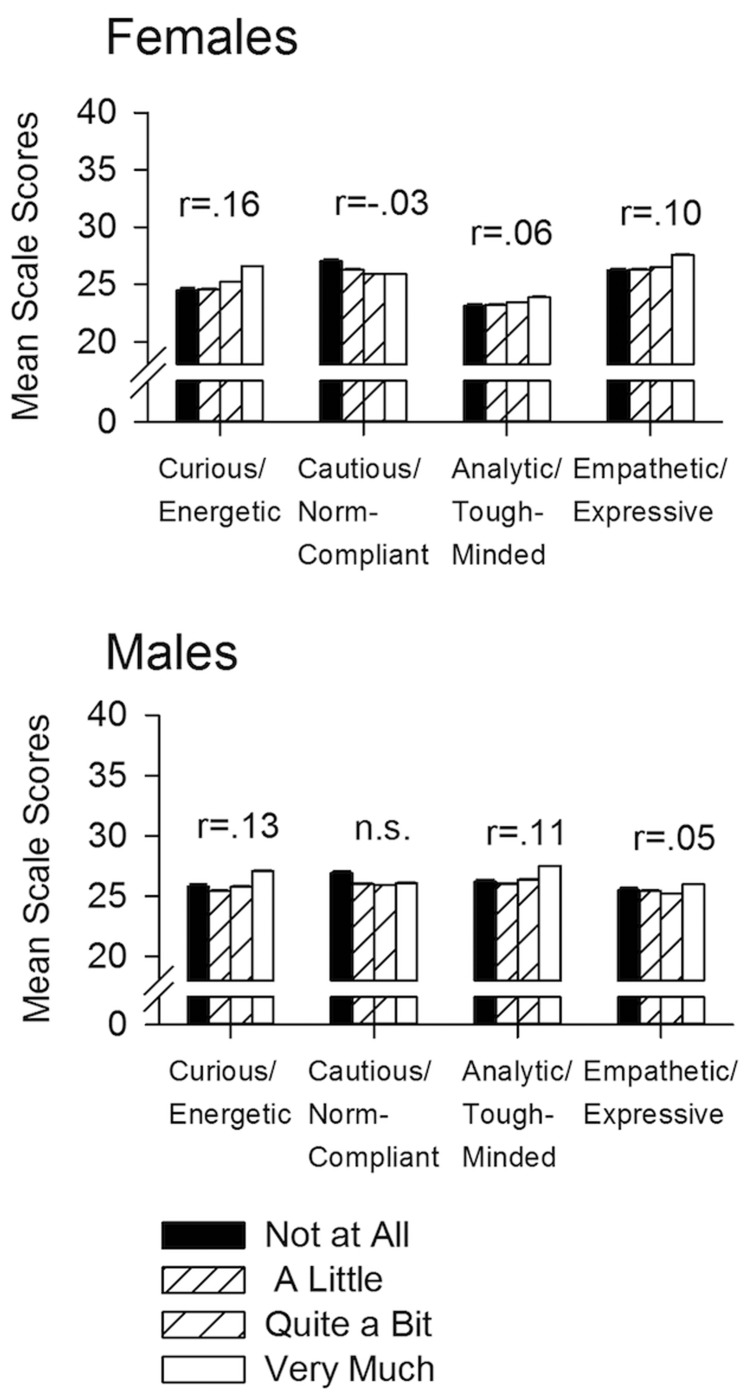
**Mean scale scores for responses to “Sex is an essential part of a successful relationship.”** The data describe attitudes about sexual activity and relationships for the four scales. For the Curious/Energetic scale, both men and women showed a positive correlation between their scale scores and level of endorsement for sexual activity in a relationship (men: *r* = 0.13, *p* = 3.89 × 10^-69^; women: *r* = 0.16, *p* < 1.0 × 10^-100^); the correlation for women was significantly higher than the one for men (*p* = 0.0024). For the Cautious/Social Norm Compliant scale, men showed no effect, while women showed a negative correlation (*r* = -0.03, *p* = 3.46 × 10^7^), which was significantly different from men (*p* = 0.0015). For the Analytic/Tough-minded scale, men showed a greater correlation than women (*r* = 0.11, *p* = 2.2 × 10^-48^ vs. *r* = 0.06, *p* < 5.19 × 10^-20^), and the two sexes were different from each other (*p* = 2.0 × 10^-19^). For the Prosocial/Empathetic scale, women showed a higher correlation than men (*r* = 0.10, *p* < 1.58 × 10^-54^ vs. *r* = 0.05, *p* = 2.09 × 10^-12^), and the sexes were different from each other (*p* = 7.2 × 10^-10^) *N* = 39,913. All effects are statistically significant using the FDR multiple comparison test at a 0.05 criterion. For SE (generally too small to see in the figure) and SD, see Results.

The Curious/Energetic scale scores and belief that “sex is essential to a successful relationship” showed the highest positive correlation among the four scales, (*r* = 0.15, *p* < 1.0 × 10^-100^, **Figure 3**); and men and women were slightly but significantly different (*p* = 0.0015). For the Cautious/Social Norm Compliant scale, women showed a negative correlation (*r* = -0.03; *p* = 3.46 × 10^7^; **Figure 3**) while men showed no correlation, and the sex difference between correlations, while slight were statistically significant (*p* = 0.0024). For the Analytical/Tough-minded scale men and women were different (*z* = 4.99, *p* = 3.02 × 10^-7^): the positive correlation was *r* = 0.11 for men (*p* = 2.2 × 10–48), compared to *r* = 0.06 for women (*p* < 5.19 × 10^-20^). The Prosocial/Empathetic scale also differed significantly by sex (*z* = 4.98, *p* = 3.18 × 10^-7^). For men, the correlation was 0.05 (*p* = 2.09 × 10^-12^), compared to.10 (*p* < 1.58 × 10^-54^) for women. However, the percent of women who answered the question “Very Much So” (45.7%) was not different from the percent of men (45.4 %; χ2 = 0.265, *p* = 0.607).

### Eigen Analysis of the FTI

**FIGURE 4:**
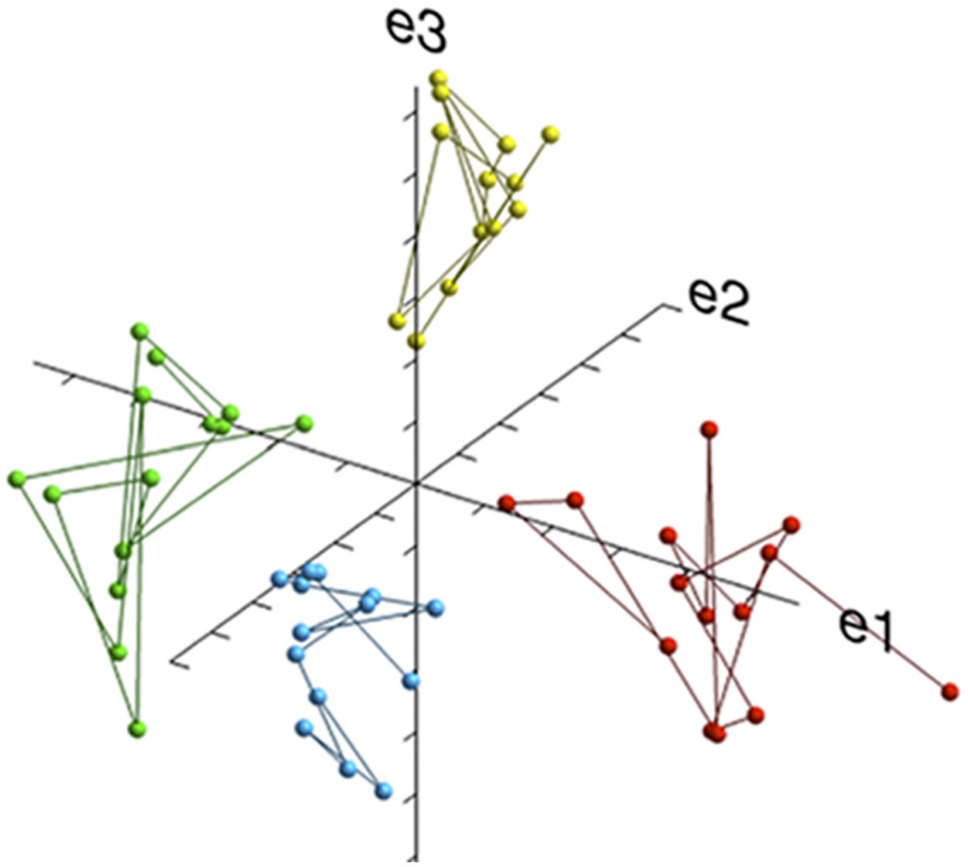
**A 3D plot of the Eigen analysis of relationships among all questions and the four major groupings of the FTI.** Each colored ball is a specific question on the FTI. Yellow, Curious/Energetic; Blue, Cautious/Social Norm Compliant; Red, Analytical/Tough-minded; Green, Prosocial/Empathetic; e = eigenvector. The position of each colored ball (in three-dimensional space) indicates the correlation coefficient calculated by the Eigen analysis for each item on the FTI. The lines connect the questions within each scale. The length of each line is a rough estimate of the covariance between items, or the eigenvector. The four scales are clearly separable.

Eigen analysis generated a set of Eigenvectors with coefficients that represent the relative positions of each item in a multi-dimensional covariance space (shown in **Figure 4**). The figure demonstrates the existence of four clusters of co-varying items associated with the four factors previously reported using factor analysis (Fisher et al., 2010b). The results were the same for the two samples of 50,000 respondents.

### Correlations between the FTI and the NEO-FFI

The Big Five has well-known value in assessing personality; and some domains of the NEO, notably Openness to Experience and Extraversion (similar to novelty-seeking) have demonstrated heritability (Jang et al., 1996). Therefore the NEO-FFI was used as our criterion to assess the convergent correlations of the four FTI domains with the five domains of the NEO-FFI. A two-tailed Pearson’s *r* correlation was conducted to determine convergent validity, as well as Cronbach’s alpha reliability analyses for an estimate of internal consistency. For this independent correlation study, a *p*-value of 0.01 was accepted.

Cronbach alphas were the same for both summed scales (0.77) and ranged from.74 to 0.84 for each scale domain (**Table 4**), indicating modest to good score reliability for both the FTI and the NEO-FFI. Significant convergent and discriminant correlations between the NEO-FFI and the FTI are provided in **Table 5**.

**Table 4:** Cronbach alpha score reliabilities for the NEO-FFI and FTI.

	Males	Females	Both	
Subscale	Mean (SD)	Mean (SD)	Mean (SD)	Alphas
NEO-FFI: Neuroticism	19.21 (7.49)	21.77 (7.06)	20.68 (7.34)	0.84
NEO-FFI: Extraversion	29.71 (7.00)	30.72 (6.10)	30.30 (6.50)	0.81
NEO-FFI: Openness	29.20 (5.47)	28.10 (5.57)	28.56 (5.54)	0.66
NEO-FFI: Agreeableness	30.67 (5.60)	33.32 (5.27)	32.21 (5.55)	0.74
NEO-FFI: Conscientiousness	31.97 (6.15)	31.77 (5.37)	31.86 (5.70)	0.78
FTI: Curious/Energetic	25.09 (4.67)	24.48 (4.24)	24.74 (4.42)	0.72
FTI: Cautious/Norm Compliant	25.86 (4.89)	26.12 (4.91)	26.01 (4.89)	0.78
FTI: Analytic/Tough-Minded	26.21 (5.04)	21.09 (4.45)	23.25 (5.34)	0.80
FTI: Prosocial/Empathetic	23.92 (5.48)	26.29 (5.50)	25.30 (5.60)	0.79

**Table 5:** Correlations between the NEO-PI and FTI subscales.

Sample type	Neuroticism	Extraversion	Openness	Agreeable	Conscientious
Curious/Energetic	-0.332^∗^	0.519^∗^	0.308^∗^	0.007	-0.018
Cautious/Norm Compliant	0.170^∗^	-0.011	-0.426^∗^	0.132	0.461^∗^
Analytic/Tough-Minded	-0.147^∗^	0.041	0.241^∗^	-0.308^∗^	0.224^∗^
Prosocial/Empathic	0.373^∗^	0.111	0.284^∗^	0.123	-0.242^∗^

*^∗^Statistically significant using FDR and *p* 0.05 criterion.*

The Curious/Energetic scale of the FTI correlated positively with the NEO-FFI domain for Openness to Experience (*r* = 0.308, *p* = 0.000015) and Extraversion (*r* = 0.519, *p* = 1.7 × 10^-19^), and negatively with Neuroticism (*r* = -0.332, *p* = 2.9 × 10^-6^).

The Cautious/Norm Compliant scale of the FTI correlated positively with the NEO-FFI domain for Conscientiousness (*r* = 0.461, *p* = 2.2 × 10^-11^) and Neuroticism (*r* = 0.17, *p* = 0.019) and negatively with the NEO-FFI domain for Openness to Experience (*r* = ^-0^.426, *p* = 8.9 × 10^-10^).

The Analytic/Tough-Minded scale of the FTI correlated negatively with the NEO-FFI scale for Agreeableness (*r* = -0.308, *p* = 0.000015). Also found was a positive correlation between Analytic/Tough-Minded and Openness to Experience (*r* = 0.241, *p* = 0.0008) and Conscientiousness (*r* = 0.224, *p* = 0.0019). There was also a negative correlation between Analytic/Tough-Minded and the NEO-FFI scale for Neuroticism (*r* = -0.147, *p* = 0.0430).

There was not a significant correlation between the Prosocial/Empathetic scale of the FTI and the NEO-FFI scale for Agreeableness (*r* = 0.123, *p* = 0.079). However, the Prosocial/Empathetic scale had positive correlations with the Neuroticism. (*r* = 0.373, *p* = 1.2 × 10^-7^), and Openness to Experience scales (*r* = 0.284, *p* = 0.0001), as well as a negative correlation with Conscientiousness (*r* = -0.242, *p* = 0.0008).

## Discussion

This investigation used several large, international samples to correlate and partially behaviorally validate the relationship between four proposed primary temperament dimensions and their proposed brain systems. The study looked at five behavioral variables, including: gender; level of education; religious preference; political orientation; and the degree to which an individual regards sex as essential to a successful relationship. We did not measure brain chemistry, but rather used behavioral characteristics correlated with brain chemistry in previous studies. Thus the results may be consistent with the overall proposed relationship between brain chemistry and the four dimensions, but they are not proof of these associations.

### Sex Differences on the Analytic/Tough-Minded and Prosocial/Empathetic scales

Males and females scored in the predicted direction for the Analytic/Tough-minded and Prosocial/Empathetic scales in North America and also in six other countries tested, including both Western and Eastern societies. Importantly, a sample from a university population (rather than a dating site) showed the same results, with even greater odds ratios for the Analytic/Tough-minded and Prosocial/Empathetic dimensions, and odds ratios closer to one for the other two dimensions. There were sex differences for some of the other scales, but these were exceptionally small (e.g., *r* = 0.004 for the Cautious/Social Norm Compliant scale in the North American sample).

These data are consistent with the hypothesis that the Analytic/Tough-minded scale measures some influence by the testosterone system, and the Prosocial/Empathetic scale measures some influence by the estrogen/oxytocin system. These data are also consistent with the results of two fMRI studies using the FTI (Brown et al., 2013). The FTI Analytical/Tough-minded scale co-varied with activity in regions of the occipital and parietal cortices associated with visual acuity and mathematical thinking, attributes linked with testosterone; testosterone also contributes to brain architecture in these areas. Further, the FTI Prosocial/Empathetic scale co-varied with activity in regions of the inferior frontal gyrus, anterior insula, and fusiform gyrus. These are regions associated with mirror neurons or empathy, a trait linked with the estrogen/oxytocin system. The effect sizes in this study were relatively small; but many other influences from biological, cultural and epigenetic forces play a role in temperament and behavior.

Interestingly, the highest percentage of Analytical/Tough-minded men and women were from Spain (47.2%; 24.8%); and the highest percentage of Prosocial/Empathic men and women were from Japan (25.8%; 52.2%; **Table 1**), even though Japan had the most men in the sample (72%). These data suggest that different cultures are composed of individuals who, collectively, express somewhat different temperament profiles, at least those who wish to find a dating partner.

### Level of Education

We predicted that Level of Education would be correlated with the Curious/Energetic scale because attaining a higher academic degree requires elevated curiosity, motivation and energy (Subotnik et al., 2011), qualities linked in the biological literature with the dopamine system (Depue and Collins, 1999; Zuckerman and Kuhlman, 2000; Wacker et al., 2006). As hypothesized, the Curious/Energetic scale showed a small but significant positive correlation with Level of Education, while the other FTI scales showed a negative correlation or minimal to no effect.

Supplementary support for this association between the Curious/Energetic scale of the FTI and the dopamine system is suggested by correlations with the NEO-FFI: We found a high correlation between the FTI Curious/Energetic scale and the Openness to Experience domain of the Big Five; the relevance of this is that the Openness domain is also positively associated with level of education and may be linked with activity in the dopamine system (DeYoung and Gray, 2009). Further, two fMRI investigations (Brown et al., 2013) have shown that higher scores on the Curious/Energetic scale co-varied with activity in brain regions linked with dopamine activity.

The above results support the hypothesis that the Curious/Energetic scale of the FTI measures, to some degree, the influence of the dopamine system.

### Religious Affiliation

Individuals scoring highest on the Cautious/Social Norm Compliant scale were significantly more likely to be members of an organized religious community. The effect size was small, but the direction of the effect was different from that of the other three scales of the FTI. These results are consistent with our hypothesis that the Cautious/Social Norm Compliant scale may measure, to some degree, serotonergic factors, because genetic data associate aspects of the serotonin system with religiosity (Lorenzi et al., 2005; Ott et al., 2005) and traditionalism (Golimbet et al., 2004).

### Political Orientation

It was predicted that participants who scored highest on the Cautious/Social Norm Compliant scale would be more politically conservative because self-reported conservatives in other western countries score higher than self-reported liberals on scales of respect for authority and tradition (Graham et al., 2009), characteristics of the Cautious/Social Norm Compliant dimension. Also, traditionalism is linked in the biological literature with aspects of the serotonin system (Golimbet et al., 2004). Consistent with the prediction, political conservatism was positively associated with high scores on the Cautious/Social Norm Compliant scale.

It was also predicted that participants who scored highest on the Prosocial/Empathetic scale would be significantly more liberal in their political views, because self-reported liberals in dozens of countries score higher than conservatives on scales of caring/nurturance (Graham et al., 2009), qualities associated in the biological literature with the estrogen and oxytocin systems (Knickmeyer et al., 2006). Consistent with the prediction, political conservatism was negatively associated with high scores on the Prosocial/Empathetic scale. These data further support other research that variability in political values is not simply attributable to differences in cognitive style, but is also, in part, associated with differences in biological factors (Alford et al., 2005; Amodio et al., 2007; Kanai et al., 2011).

### Sex as Essential to a Relationship

It was predicted that scores on both the Analytical/Tough-minded scale and the Curious/Energetic scale would positively correlate with the statement, “Sex is an essential part of a successful relationship” because elevated activity in the testosterone and dopamine systems is widely associated with elevated sex drive (Bagatell et al., 1994; Meston and Frohlic, 2000) and we reasoned that those individuals with a higher sex drive would be more likely to regard sex as important to a successful partnership. These predictions were supported.

Further, since higher central serotonin regularly suppresses sexual desire and sexual function (Rosen et al., 1999), we also predicted that higher scores on the Cautious/Social Norm Compliant scale would negatively correlate with the statement, “Sex is an essential part of a successful relationship,” because individuals with a lower sex drive might regard sex as less important to a successful partnership. Scores on the Cautious/Social Norm-Compliant scale did show a negative correlation with the statement, “Sex is an essential part of a successful relationship.”

### Comparison with the NEO-FFI

We compared responses on the FTI with those on the NEO-FFI (the shortened form of the NEO-Personality Inventory; Costa and McCrae, 1992), not only to assess the criterion validity of the FTI using an established measure; but also to further explore the potential characteristics linked with the FTI scales. Our three predictions were supported. Moreover, this comparison suggested several qualities associated with the FTI that we had not previously associated with this measure.

#### Curious/Energetic Scale

The Openness to Experience domain of the NEO-FFI and the Curious/Energetic scale of the FTI were positively correlated (*r* = 0.308, *p* = 0.000015). As both attempt to measure exploratory behavior, novelty-seeking and curiosity (Costa and McCrae, 1992; Depue and Collins, 1999), this positive correlation was anticipated. Interestingly, the Openness to Experience domain of the NEO-FFI is also the only domain of the Big Five that has shown a consistent, positive correlation with general intelligence (DeYoung et al., 2005), while the Curious/Energetic scale of the FTI is positively correlated with level of education. This suggests convergent data for these two dimensions. But it also suggests that the Curious/Energetic scale of the FTI may measure some aspect of general intelligence, as well as level of education.

The Extraversion scale of the NEO-FFI and the Curious/Energetic scale on the FTI represented the strongest positive correlation between the two measures (*r* = 0.519, *p* = 1.7 × 10^-19^). Perhaps because the Extraversion domain of the NEO-FFI is associated with risk-taking and energy (Depue and Collins, 1999), consistent with the dopamine system (Cohen et al., 2005; DeYoung and Gray, 2009), these qualities are consistent with those of the Curious/Energetic scale on the FTI.

The Curious/Energetic Scale demonstrates convergent validity with the NEO-FFI domains of Openness to Experience and Extraversion. This is meaningful, as Extraversion scores have been positively correlated with the volume in the medial orbitofrontal cortex (Omura et al., 2005; Rauch et al., 2005), a brain area associated with coding the hedonic value of reward (DeYoung et al., 2010). While the Openness to Experience domain has been positively correlated with parietal areas predictive of working memory and the control of attention (DeYoung et al., 2010), it is also the only Big Five trait associated with intelligence (DeYoung et al., 2005). The Curious/Energetic scale of the FTI is positively correlated with the substantia nigra (Brown et al., 2013), an important brain area involved in the reward path, and is significantly correlated education level. These data suggest that high scores on the Curious/Energetic scale of the FTI may measure some form of Extraversion and Openness/Intellect.

#### Cautious/Social Norm Compliant Scale

It was anticipated that scores on the Cautious/Social Norm Compliant scale of the FTI would correlate with the Conscientious scale of the NEO-FFI because both the NEO-FFI domain of Conscientiousness and the Cautious/Social Norm Compliant scale on the FTI attempt to measure self-control and self-regulation (Costa and McCrae, 1992), as well as the desire to plan and organize (DeYoung and Gray, 2009). These two scales were significantly correlated in a positive direction (*r* = 0.461, *p* = 2.2 × 10^-11^), showing convergence. Additionally, a positive correlation was found between the FTI Cautious/Social Norm Compliant scale and the Neuroticism scale of the NEO-FFI (*r* = 0.170, *p* = 0.019), perhaps suggesting that caution and the desire to conform to social rules can be linked with anxiety in social situations.

#### Analytic/Tough-minded Scale

The prediction that higher scores on the Analytical/Tough-minded scale of the FTI would correlate negatively with high scores on the Agreeableness scale of the NEO-FFI was supported. We anticipated this relationship because tough-mindedness is likely to be the opposite of tender-mindedness, a trait in the Agreeableness domain of the NEO-FFI. There was, however, an unanticipated positive correlation between the Analytic/Tough-minded scale of the FTI and the NEO-FFI domain for Conscientiousness (*r* = 0.224, *p* = 0.0019). Perhaps this correlation is indicative of a mutual sense of purpose, determination, attention to detail and will to achieve (Costa and McCrae, 1992). The unanticipated positive correlation found between the Analytic/Tough-minded scale of the FTI and the NEO-FFI scale for Openness to Experience (*r* = 0.241, *p* = 0.0008) may also derive from these shared attributes.

#### Prosocial/Empathic Scale

Consistent with the literature (McCrae et al., 2000; Costa et al., 2001; Chapman et al., 2007), women scored higher on the NEO-FFI domains of Neuroticism and Agreeableness. They also scored higher on the Prosocial/Empathic scale of the FTI than the men (*r* = 0.373, *p* = 1.2 × 10^-7^).

In contrast to our prediction that Agreeableness and the Prosocial/Empathic scale of the FTI would be positively correlated, there was not a significant relationship. This scale divergence is interesting since Agreeableness is essentially the prosocial domain of the NEO. Though Agreeableness is not associated with empathy in the NEO, it does measure compliance, trust, modesty, tolerance and tender-mindedness (Costa and McCrae, 1992). In fact, in a recent study of personality and brain activity during emotional attribution decisions, participants with higher Agreeableness scores also showed greater right temporoparietal junction activity, a brain region associated with perspective-taking and Theory of Mind (Haas et al., 2015), qualities thought to contribute to the empathy. However, since empathy was not formerly associated with Agreeableness, the HEXACO personality model included a facet called Emotionality to specifically address empathy, attachment, and harm-avoidance (Ashton and Lee, 2007). Further, when the FTI was administered as part of two fMRI studies (Brown et al., 2013), participants with higher scores on the Prosocial/Empathic scale showed greater activity in the inferior frontal gyrus, anterior insula and fusiform gyrus, regions associated with estrogen binding and empathic behavior, suggesting that the Prosocial/Empathic scale does measure qualities of the domain of Agreeableness associated with the NEO and the empathy/attachment measure of Emotionality in the HEXACO.

Last, the Prosocial/Empathetic scale of the FTI was positively correlated with the NEO-FFI scale of Openness to Experience (*r* = 0.284, *p* = 0.0001) and negatively correlated with the NEO-FFI scale for Conscientiousness (*r* = -0.242, *p* = 0.0008).

### Novel Aspects and Potential Advantages of the FTI

The FTI was not developed to replace other measures of personality. It does not measure neuroticism or extraversion, for example. But based on the results of our convergent and discriminant analyses, the modest length of the FTI and its additional constructs of empathy, tough-mindedness and degree to which one regards sex as essential to a partnership, the FTI may be a useful complement to the NEO-FFI or other Five Factor Models of personality.

The novel value of the 56-item FTI within a business or organizational context may be to highlight individual differences in style of communication, style of leadership, preference for rules and schedules, attitude toward risk, tendency to trust, sensitivity to rank, degree of emotional containment, tendency toward traditionalism, degree of linguistic and/or mathematical creativity, and proficiency at executive social skills. The potential value of the FTI in a personal context may be to lend additional insight into attitudes of friends, partners, and kin regarding their political and religious presuppositions, their educational aspirations, and their views regarding the importance of sex to a relationship (an important component of partnership viability) and partner–partner and parent–child compatibility. The potential value of the FTI to the science of personality is that it is derived directly from brain architecture and physiology, providing an additional way to look at the core structure of temperament. Last, this additional approach may be able to simplify temperament explanations and uses. For example, with the rationale that dopamine and its receptors strongly influence behavior, some of the domains from linguistically derived questionnaires like the BFI that uses Extraversion and Openness to Experience might be collapsed into one domain and thus simplified. Thus, physiology and behavior based on hormonal and neurotransmitter influences may be able to cover a broader spectrum than several other constructs. In short, the FTI may provide a parsimonious construct.

Anecdotal evidence suggests that the FTI is useful in a variety of spheres. A public service group has initiated a project that uses the FTI to match foster parents with foster children; a major American accounting firm has used the FTI to train 45,000 employees on how to structure conversations and presentations with potential clients. The largest international Internet dating service is using the FTI to enable members to better understand their likely compatibility with potential life partners; currently 14 million men and women in 40 countries have taken the questionnaire for purposes of insight. A major international credit card company has used the FTI to further understand their card users; and couples therapists are using the FTI to enable couples to understand their differences and solve ingrained issues. These users have anecdotally reported (to HEF) that the FTI is easy to explain, understand, and apply.

### Limitations

The functional significance of the statistically significant but small effect sizes is yet to be determined. These quantitative differences may not translate into relevant behavioral differences between individuals or groups. Conversely, these small effect sizes may be an accurate representation of these four biological systems, largely because these systems are subject to many physiological interactions with one another, with other biological systems, and with social and epigenetic forces that contribute to phenotypic variations in temperament. Moreover, other studies show very small size effects and suggest that the small effect sizes reported in this paper are appropriate and could be meaningful (de Moor et al., 2010).

Further, it has been argued that almost any data will be significant using a large sample. But statistically significant differences are not inevitable with large samples. They only appear if there is an effect in the population, and they indicate that the effect would still be found with replication. Large samples provide the opportunity to find small but significant effects that normally would be overwhelmed by statistical noise. In fact, small effect sizes are not unusual for studies of large populations (de Moor et al., 2010).

Another limitation is that for the analyses, random samples of the population were not used; instead, the samples were largely based on unmarried individuals who were looking for a partner, who had access to a computer, who were willing to pay to join an Internet dating site, and who felt comfortable using an online dating service. This is why it was important that the basic sex findings were replicated in a university sample as well.

However, the Internet population we tested represents a significant and important group. Over one–third of the adult U.S. population is single (over 100 million individuals); and with a current divorce rate exceeding 45%, almost half of Americans have been or are likely to become single at some point in their lives (Taylor et al., 2011). The populations examined in this study represent a large and growing percentage of the broad U.S. population and those of several other countries. Subjects also ranged in age from 18 to 88 years; they were from every major ethnic group (e.g., European American, African American, Asian American, and Latino); they lived in rural, suburban and urban areas; and they resided in all 50 states in the U.S., as well as in Canada and six additional cultures, both Eastern and Western.

Last, participants may have skewed their responses to enhance their social desirability. However, participants responding to any questionnaire that uses self-appraisals will approach the task with an array of subliminal and cognitive agendas that cannot be fully screened. In fact, the correlation analyses and the Eigen analysis of the FTI samples are more comprehensive than the samples used in most psychological studies that canvas the attitudes and behaviors of college populations paying a large fee for college entrance, coming largely from similar backgrounds, of the same general age, and sharing similar life styles and life goals.

### Future Directions

To further explore the FTI measure, an investigation is underway to assess the relationship between 63 specific alleles and the four FTI temperament dimensions. The essential study of test–retest reliability of the FTI is in preparation as well. To apply these data to life situations, we examined the role of these proposed temperament dimensions in initial mate choice (Fisher, 2009; Fisher et al., 2010b); this investigation continues. Further research could also explore how these four broad proposed styles of thinking and behaving effect one’s proneness to divorce, adultery, and other social, reproductive, cognitive, affective and/or motivational processes, as well as their varying expression in different cultures, different age groups, different occupations, and among those of different sexual orientations and those with different medical conditions.

One promising field for future investigation may be exploration of the possible relationship between these temperament dimensions and specific psychiatric diseases, due to accumulating data associating several psychiatric syndromes with specific neural substrates. For example, perhaps individuals primarily expressive of the Curious/Energetic scale are disproportionately susceptible to substance abuse, because several of the primary addictions are linked with activity in the mesolimbic dopamine system (Fowles, 2001; Dawe et al., 2004; Loxton et al., 2011). They may also be predisposed to diseases linked with mania, including bipolar affective disorder and the schizophrenia spectrum. These diseases have been linked with alterations in the activities of the catecholamines (Kapura et al., 2005; Dalley and Roiser, 2012; Kang et al., 2013) and dopamine antagonists reduce some of the symptoms of these conditions (Ginovart, 2012). Also, traits associated with types of Attention Deficit Hyperactivity Disorder (ADHD) have been linked with imbalances in the dopamine and norepinephrine systems (Zametkin, 1987), as well as a specific allele in the dopamine receptor D4 gene (Faraone et al., 2001). The testosterone system has been associated with diseases in the Autism Spectrum (Geschwind and Galaburda, 1985; Baron-Cohen and Hammer, 1997; Baron-Cohen et al., 2005), so those expressive of the Analytical/Tough-minded scale may be predisposed to these. The testosterone system is also associated with aggressiveness, so individuals expressive of this temperament dimension may be disproportionately susceptible to violent or anti-social behavior (Nyborg, 1994). Last, activity in the estrogen system is commonly linked with clinical depression (Stahl, 1998), perhaps predisposing those expressive of the Prosocial/Empathetic scale of the FTI to anxiety and depression.

Regardless of the many studies linking aspects of various diseases with neural systems, no single neurotransmitter or hormone system is likely to be responsible for the full array of symptoms in any disease pattern. Instead, a multitude of factors influence how each of these neural systems impact one another, affect other neural systems, modifiers and genomic activational events, and contribute to cognitive and behavioral outcomes. Much further investigation is necessary to establish substantive links between the temperament dimensions of the FTI and specific bio-behavioral illnesses.

## Conclusion

The FTI is, to our knowledge, the first measure of temperament constructed directly from brain science, using four basic neuromodulator systems, that was *subsequently* tested and partially validated by two fMRI brain-scanning studies, rather than finding physiological correlates for proposed traits established by other means. This approach may produce broader, more useful temperament dimensions for further study because they are less likely to show trait crossovers, physiologically, than The Big Five, for example. The Curious/Energetic scale may subsume both Openness to Experience and Extraversion. In addition, the model is a clearly testable hypothesis. Further, the correlations of the FTI temperament dimensions with five behavioral variables, as well convergent and discriminant validity with the NEO-FFI, give us reason to suggest that the FTI may be useful in psychotherapy, business, medicine, and the legal community to understand and serve individuals with different temperament profiles. It was designed to be a complement to existing measures and may be most useful for informing users about compatibility between individuals in all aspects of life, from household to work environments. The FTI may have broad applications, as well as initiate several further lines of inquiry into the on-going investigation of the biological structures of personality.

## Conflict of Interest Statement

The authors declare that the research was conducted in the absence of any commercial or financial relationships that could be construed as a potential conflict of interest.
